# A spinach genome assembly with remarkable completeness, and its use for rapid identification of candidate genes for agronomic traits

**DOI:** 10.1093/dnares/dsab004

**Published:** 2021-06-17

**Authors:** Hideki Hirakawa, Atsushi Toyoda, Takehiko Itoh, Yutaka Suzuki, Atsushi J Nagano, Suguru Sugiyama, Yasuyuki Onodera

**Affiliations:** 1 The Department of Technology Development, Kazusa DNA Research Institute, Kisarazu, Chiba 292–0818, Japan; 2 Department of Genomics and Evolutionary Biology, National Institute of Genetics, Mishima 411-8540, Japan; 3 School of Life Science and Technology, Tokyo Institute of Technology, Meguro‐ku, Tokyo 152‐8550, Japan; 4 The Department of Computational Biology and Medical Sciences, Graduate School of Frontier Sciences, The University of Tokyo, Kashiwa 277–8568, Japan; 5 Faculty of Agriculture, Ryukoku University, Otsu, Shiga 520-2194, Japan; 6 School of Agriculture, Hokkaido University, Sapporo 060–8589, Japan; 7 The Research Faculty of Agriculture, Hokkaido University, Sapporo 060–8589, Japan

**Keywords:** spinach, resistance gene analogues, bolting timing, fruit/seed shape

## Abstract

Spinach (*Spinacia oleracea*) is grown as a nutritious leafy vegetable worldwide. To accelerate spinach breeding efficiency, a high-quality reference genome sequence with great completeness and continuity is needed as a basic infrastructure. Here, we used long-read and linked-read technologies to construct a *de novo* spinach genome assembly, designated SOL_r1.1, which was comprised of 287 scaffolds (total size: 935.7 Mb; N_50_ = 11.3 Mb) with a low proportion of undetermined nucleotides (Ns = 0.34%) and with high gene completeness (BUSCO complete 96.9%). A genome-wide survey of resistance gene analogues identified 695 genes encoding nucleotide-binding site domains, receptor-like protein kinases, receptor-like proteins and transmembrane-coiled coil domains. Based on a high-density double-digest restriction-site associated DNA sequencing-based linkage map, the genome assembly was anchored to six pseudomolecules representing ∼73.5% of the whole genome assembly. In addition, we used SOL_r1.1 to identify quantitative trait loci for bolting timing and fruit/seed shape, which harbour biologically plausible candidate genes, such as homologues of the *FLOWERING LOCUS T* and *EPIDERMAL PATTERNING FACTOR-LIKE* genes. The new genome assembly, SOL_r1.1, will serve as a useful resource for identifying loci associated with important agronomic traits and for developing molecular markers for spinach breeding/selection programs.

## 1. Introduction

Spinach (*Spinacia oleracea*) is a major crop in the goosefoot family (Chenopodiaceae s.s.), along with sugar beet (*Beta vulgaris*) and quinoa (*Chenopodium quinoa*). Unlike most members of the goosefoot family (*x *=* *9), spinach is dioecious, and has *n *=* x *=* *6 chromosomes including sex chromosomes (XY),^1^ which are numbered 1–6 according to the nomenclature system defined by Sugiyama and Suto.^2^ The genome size of spinach was estimated as 989 Mb, based on the C-value.^3^ Recently, the draft genome sequence of the spinach line Sp75 was determined; this sequence has a total size of 996 Mb with a scaffold N_50_ size of 2.2 Mb, nearly equal to the genome size estimated based on the C-value.^4^ The draft genome was shown to have a high content (>70%) of transposable elements (TEs), among which *Copia* and *Gypsy* retroelements are predominant, as in many plant genomes.^5^^,^^6^ Although very informative and useful, the genome assembly remains highly fragmented (77,269 sequences) and contains a significant proportion (16.6%) of sequencing gaps filled with undetermined nucleotides (Ns).^4^ Furthermore, the six pseudomolecules represent only half of the genome assembly. Although repetitive, heterozygous sequences or biased read coverages, etc., can be considered as causes of this problem; the main cause may be the highly repetitive nature of the spinach genome, as mentioned above: sequencing reads which are shorter than repetitive sequences result in fragmented genome assemblies.^7^^,^^8^

The goals of spinach breeding programs include controlling sexual phenotype, the timing of bolting (initiation of the flowering stem), fruit/seed shape, and leaf morphology, achieving resistance to pests and diseases, and improving the crop yield and quality.^9^^,^^10^ Spinach is known as a dioecious species, but certain varieties, lines and populations produce monoecious plants bearing both pistillate and staminate flowers. The dioecism and monoecism are relevant to the production of spinach hybrid seeds, and elucidation of the mechanisms underlying sex determination and development of sex-diagnostic markers are valuable undertakings for spinach breeding programs.^11^^,^^12^

The timing of bolting/flowering is a crucial trait determining the suitable regions and seasons for growing this crop. The central genetic components of the flowering pathways share certain common features among divergent lineages of flowering plants, but are not always rigorously conserved.^13–15^ In many *Brassicaceae*, the vernalization requirement is mediated by a flowering repressor known as the *FLOWERING LOCUS C* (*FLC*) gene or by an *FLC* orthologue,^16^ whereas in sugar beet, the *FLC* homolog does not play a major role in the vernalization pathway.^17^ Furthermore, sugar beet does not harbour a true orthologue of the *Arabidopsis CONSTANS* (*CO*) gene, which acts upstream of *FLOWERING LOCUS T* (*FT*) in the photoperiod pathway.^18^^,^^19^ However, it remains uncertain whether the genetic components of the flowering pathways are highly conserved between spinach and sugar beet, which belong to different subfamilies (Chenopodioideae and Betoideae) that are not closely related within the goosefoot family.

Most recent spinach varieties produce round seeds (fruits), while certain traditional varieties and spinach wild relatives, i.e. *Spinaciaturkestanica* and *Spinaciatetrandra*, bear prickly seeds (fruits).^10^^,^^20^ The round seed is a preferable form for spinach breeding, since it is easy to handle, and very well suited for seed processing and machine sowing [personal communication from Tohoku seed Co. (Utsunomiya, Japan)]. The prickly seeds are considered to have ‘primitive’ (ancestral) characteristics, and round seeds are thought to be associated with domestication.^21^ The prickles are formed on pseudocarps, and their development was shown to be controlled by a single dominant factor.^22^^,^^23^ However, nothing is known about the molecular basis for the mechanism of prickle development in spinach.

Fungal and viral pathogens such as *Peronospora effusa* (downy mildew), *Fusarium oxysporum* (fusarium wilt), *Albugo occidentalis* (white rust), and cucumber mosaic virus (blight) cause serious damage to spinach production.^24^ Downy mildew in particular is a major fungal disease of spinach worldwide. The genes encoding a receptor-like protein (RLP) and nucleotide-binding site/leucine-rich repeat domains (NBS-LRRs) were recently identified as the candidates for the downy mildew resistance gene, *RPF1*, in spinach.^25^ Spinach germplasm and varieties with resistance/tolerance to these fungal and viral diseases have been found, but with the exception of the gene for downy mildew resistance, the responsible genes have not yet been identified [personal communication from Tohoku seed Co. (Utsunomiya, Japan)].

Marker-assisted selection would significantly improve the efficiency of breeding programs by enabling accurate selection for traits of interest using highly reliable markers. Mapping studies have identified DNA variants linked to or associated with critical traits in spinach breeding programs.^4^^,^^26^^,^^27^ These variants may provide clues for the development of selection markers. Identification of loci and variants responsible for agronomic traits and discovery of the allelic variations would be necessary for the development of highly reliable and practically useful selection markers that could detect variants tightly linked to or directly responsible for desirable traits. In this context, a high-quality reference spinach genome which comprehensively and accurately captures the genomic landscape would provide a solid foundation to accelerate genetic mapping and identification of genetic variants, and would also be a valuable resource for spinach breeding programs.

Here, we undertook *de novo* sequencing of the spinach genome by using different long-read sequencing technologies and a linked-read sequencing technique, and generated a high-quality genome assembly, designated SOL_r1.1 (total size: 935.7 Mb; N_50_ = 11.3 Mb). Furthermore, to establish a foundation for identifying candidates for disease-resistance genes, we carried out a genome-wide survey of resistance gene analogues (RGAs), such as NBS-encoding proteins, receptor-like protein kinases (RLKs), and RLPs, which are potentially associated with pathogen-associated molecular pattern-triggered and effector-triggered immunity systems.^28^ We also conducted double-digest restriction-site-associated DNA sequencing (ddRAD-seq)^29^ of a spinach F_2_ population and quantitative trait locus (QTL) analysis of bolting timing and seed/fruit shape, to construct a fine molecular linkage map and pseudomolecules representing the six spinach chromosomes, and to evaluate the reliability and usefulness of the genome annotation for SOL_r1.1 and the resultant linkage map.

## 2. Materials and methods

### 2.1. Plant materials

The plants used to prepare total cellular DNA for the short-, long-, and linked-read sequencing analyses were cultivated as follows. Plants from the dioecious breeding line 03-009 (Tohoku Seed, Utsunomiya, Japan)^30^ were grown in a growth chamber (LH-350S; Nippon Medical & Chemical Instruments, Osaka, Japan) at 20°C under an 8-h photoperiod for two months. A spinach F_2_ population was produced by a cross between a female plant of the line 03-009 and a plant from the monoecious line 03-336 (Tohoku Seed)^30^ and was used for linkage analysis and QTL mapping study. Plants from this population were grown in a growth chamber (LH-350S; Nippon Medical & Chemical Instruments) at 20°C with an 8 h photoperiod for the first two months, at 22.5°C with a 16 h photoperiod over the next one week, and then at 25°C with a 24-h photoperiod. In this study, the onset of bolting was defined as the day when the main stem bolted 1 cm.

### 2.2. DNA sequencing

The total cellular DNA used for the short-, long-, and linked-read sequencing was extracted from young leaves harvested from a single male and single female plant of line 03-009, and used for library preparation. One of two long-read sequencing libraries was sequenced using a Sequel II system (Pacific Biosciences, Menlo Park, CA), and the other one was run on the MinION and GridION flowcells (Oxford Nanopore Technologies, Oxford, UK; Supplementary Table S1). The paired-end (PE), mate-pair (MP) and the linked-read library were run on a HiSeq 2500 (Illumina; Supplementary Table S2). Total cellular DNA subjected to the ddRAD-Seq^29^ was extracted from F_2_ and F_1_ plants from a 03-009 × 03-336 cross and their parents (03-009 and 03-336). A total of 113 ddRAD-seq libraries was constructed by employing the method of Peterson et al.^29^ with the modifications made by Sakaguchi et al.^31^ Each F_2_ plant was included in one library (101 F_2_ plants), and the F_1_ plant and its parents were included in twelve libraries (four libraries per plant) to obtain sufficient read depth. The libraries were sequenced using a HiSeq X Instrument (Illumina) (Supplementary Table S3).

### 2.3. *De novo* genome assembly and gene annotation

The genome size of line 03-009 was estimated using Illumina PE reads by Jellyfish v2.2.6.^32^ The PacBio Sequel long-reads were assembled by FALCON v2017.11.02-16.04^33^ with default parameters, and the resultant primary contigs and associated contigs were applied to FALCON-Unzip^33^ for phasing. The primary contigs and haplotigs were polished by Sequel reads and Illumina PE reads by Arrow (https://github.com/PacificBiosciences/GenomicConsensus (17 May 2021, date last accessed)) and Pilon v1.23,^34^ respectively. The primary contigs were connected with Illumina MP reads by Opera-LG v2.0.6.^35^ Detected mis-assemblies were corrected with 10x Genomics linked-reads by Tigmint v1.1.2,^36^ and the resultant scaffolds were connected by ARKS v1.0.4.^37^ Finally, the scaffolds were further connected with Nanopore long-reads by Opera-LG,^35^ and the resultant genome assembly was designated SOL_r1.0.

The genes were predicted by BRAKER2 v2.1.0 with RNA-Seq data (Supplementary Table S4). The genes having the highest scores were selected from the splicing variants obtained by the gene prediction. The genes were searched against the UniProtKB database (https://www.uniprot.org (17 May 2021, date last accessed)) with *E*-value ≤ 1e-10 and identity ≥ 98% and NCBI’s NR database (ftp://ftp.ncbi.nlm.nih.gov/blast/db/FASTA/) with *E*-value ≤ 1e-10 and identity ≥ 95% by DIAMOND v0.9.29.130^38^ in ‘more-sensitive’ mode, respectively. The genes were searched against the protein sequences (spinach_pep_v1) of *S.oleracea* Sp75 released from SpinachBase (http://spinachbase.org (17 May 2021, date last accessed)) by BLAST+ with *E*-value 1e-50 and 90% ≤ length coverage ≤ 110% and identity ≥ 98% and identity in high scoring pairs ≥98%. The genes were also searched against the Pfam v33.1^39^ database with *E*-value ≤ 1e-80 by HMMER v3.2.1.^40^ The RNA-Seq reads were mapped against the genes by Salmon v1.2.1,^41^ and the transcripts per million (TPM) value was calculated. The predicted genes were classified into three categories, high confidence (HC), low confidence (LC), and TEs. The genes having hits in these searches and those with TPM values > 0.0 considered to be expressed were classified into the HC category. The genes with TPM value = 0.0, and those with hits or no-hits against UniProtKB, NR, and homologues of Sp75, and against the protein domains of Pfam in the criteria described above were classified into the LC category. The genes having hits against UniProtKB with the keywords related to TEs were classified into the TE category. The protein sequences of the genes (HC) of *S. oleracea* line 03-009 were compared with those of *S. oleracea* Sp75 and Viroflay, as well as *B. vulgaris*^42^ and *C. quinoa*,^43^ by OrthoFinder v2.1.2.^44^

### 2.4. Repetitive sequences

The repetitive sequences were detected *de novo* by using RepeatModeler v1.0.11 (http://www.repeatmasker.org/RepeatModeler/ (17 May 2021, date last accessed)). The repetitive sequences were searched against Repbase^45^ by RepeatMasker v4.0.7 (http://www.repeatmasker.org (17 May 2021, date last accessed)). Finally, the repetitive sequences detected in the assembled genome sequence were masked with small characters as softmasked by RepeatMasker. The two kinds of repetitive sequence, *SpRE1* and *SpRE2*, which were previously identified as *Copia*-like and its derivative elements, respectively,^46^ were also detected by RepeatMasker.

### 2.5. Inference of RGAs

The RGAs encoding NBSs, RLKs, RLPs, and transmembrane-coiled coil (TM-CC) domains, which are considered candidates of R-genes, were searched by using RGAugury.^28^

### 2.6. Construction of linkage map and QTL analysis

The quality-filtered RAD-seq reads were mapped to the spinach genome assembly using the Bowtie2 v2.3.5.1 program.^47^ The SNPs were filtered by using VCFtools v0.1.16^48^ (https://vcftools.github.io/index.html (17 May 2021, date last accessed)), based on ‘stringent’ (–max-missing 0.95 –minQ 20 –minDP 20 –maxDP 200 –maf 0.025 –min-alleles 2 –max-alleles 2 –remove-indels) and ‘less stringent’ (–max-missing 0.80 –minQ 20 –minDP 10 –maf 0.025 –min-alleles 2 –max-alleles 2 –remove-indels) criteria for quality/depth of mapped reads, and further filtered based on genotypes of the parental and F_1_ plants (homozygous in both parents and heterozygous in the F_1_ plant) by using SnpEff v4.3t^49^ (https://pcingola.github.io/SnpEff/ (17 May 2021, date last accessed)). The Lep-Map3 v0.2^50^ was used to construct a draft linkage map, which was verified and corrected by the plotRF function in the R/qtl v1.46-2^51^ (https://rqtl.org (17 May 2021, date last accessed)). QTL analysis was conducted by the composite interval mapping procedure implemented in R/qtl.

## 3. Results and discussion

### 3.1. *De novo* genome assembly

The k-mer frequency plot made by using Illumina PE reads is shown in Supplementary Fig. S1. According to the plot, the genome size of line 03-009 was estimated to be 1.05 Gb, which is nearly equal to the previously reported values, 1,009  and 989 Mb, estimated based on the same method^4^ and flow cytometry,^3^ respectively. The eight cells of PacBio Sequel reads were subjected to *de novo* assembly by FALCON, and 1,015 primary contigs (total length: 946.3 Mb; N50 length: 6.4 Mb) and 1,201 associate contigs (total length: 144.9 Mb; N50 length: 156.7 kb) were constructed (Supplementary Table S5a). The primary contigs and associate contigs were phased by FALCON-Unzip, and 550 primary contigs (total length: 933.2 Mb; N50 length: 6.7 Mb) and 6,750 haplotigs were constructed (Supplementary Table S5b). The primary contigs were polished with PacBio Sequel reads (Supplementary Table S5c), and then they were scaffolded with Illumina MP reads (Supplementary Table S5d). The mis-assemblies were corrected with 10× Genomics linked-reads by Tigmint (Supplementary Table S5e) and scaffolded with 10× Genomics linked-reads by ARKS, and then 573 primary contigs (total length: 934.4 Mb; N50 length: 8.5 Mb) were constructed (Supplementary Table S5f). Next, the primary contigs were scaffolded with Nanopore long-reads using Opera-LG and polished with Illumina PE reads, and then the initial genome assembly (SOL_r1.0; Supplementary Table S5g) composed of 287 primary contigs (total length: 935.7 Mb; N50 length: 11.3 Mb) was constructed.

Illumina short reads obtained from a single male and single female plant were mapped to SOL_r1.0, and the ratio (M/F ratio) of the mapped read coverage of male to female onto scaffolds of the assembly was computed and used to identify sex-linked (Y-linked) regions: more reads from male (XY) than female (XX) tended to be mapped to the Y-linked region, which does not recombine with the X chromosome and should be divergent from the X-linked region. Finally, the definitive genome assembly, designated SOL_r1.1 (GenBank accession numbers: BOUE01000001-BOUE01000287; Supplementary Table S5h), representing a female (XX) genome was generated by filtering out scaffolds [log10 (M/F ratio) > 0.2, scaffold size > 10 kb] representing Y-linked regions from SOL_r1.0.

As shown in Supplementary Table S5, SOL_r1.1 was found to have a high BUSCO score (96.9%), which is almost the same as those of the previously published spinach genome assemblies, Sp75 v1.0 (96.7%) and Viroflay v1.0.1 (91.5%). However, when each of the assembled sequences was assessed based on the LTR Assembly Index (LAI),^52^ which evaluates assembly continuity using long terminal repeat retrotransposons (LTR-RTs), SOL_r1.1 was found to have an LAI of 15.91, which is a ‘Reference’ grade quality (10 ≤ LAI < 20) and much higher than those of Sp75 v1.0 (LAI = 1.13) and Viroflay v1.0.1 (LAI = 1.47). The results may suggest that, in our study, repetitive sequences such as LTR retrotransposons were correctly determined by long-read sequencers and successfully assembled into scaffolds, and that among the three genome assemblies there is little difference in completeness of the gene‐rich euchromatic regions.

### 3.2. Repetitive sequences

The repetitive sequences detected in SOL_r1.1 (03-009) and the other spinach lines, Sp75 and Viroflay, are summarized in Supplementary Table S6. The repetitive sequences classified into *Copia* were most widely distributed (25.8%) in SOL_r1.1. On the other hand, the numbers of repetitive sequences of *Copia* in Sp75 and Viroflay were slightly smaller than the numbers in *Gypsy*. The subtotal of the known repeats in SOL_r1.1 (528 Mb) was greater than those in Sp75 (362 Mb) and Viroflay (107 Mb), which may indicate that the repetitive sequences classified into *Gypsy* were correctly sequenced because the genome assembly of SOL_r1.1 was performed based on the long-read type sequencer in SOL_r1.1. The number of unique repetitive sequences in SOL_r1.1 (138 Mb) was smaller than that in Sp75 (223 Mb) and was almost the same as that in Viroflay (139 Mb). As a result, the percentage of the total repetitive sequences of SOL_r1.1 was 71.2%, and this was the largest value among the three spinaches (Sp75: 58.7%; Viroflay: 49.4%). Just as in the case of assessment based on the LAI, these results indicated that the completeness of the genome sequence of SOL_r1.1 was higher than those of Sp75 and Viroflay.

### 3.3. Gene prediction and annotation

A total of 130,129 genes were initially predicted from the softmasked SOL_r1.1 scaffolds (see Materials and Methods) by BRAKER2 using RNA-Seq data. The 53,035 genes (hereafter referred to as ‘best’ genes) having the highest score among the splicing variants were selected (Supplementary Table S7). The ‘best’ genes were conducted to the DIAMOND searches in more sensitive mode against UniProtKB and NCBI NR databases, BLAST+ searches against the protein sequences of *S. oleracea* Sp75, and domain searches against Pfam database. The 23,657 genes with TPM values > 0 calculated by Salmon were estimated as being expressed. The ‘best’ genes were classified into the HC, LC, and TE categories according to the similarity searches and expression values described above, and the categorized gene sets were named SOL_r1.1a and made publicly available in the Spinach Genome DataBase (http://spinach.kazusa.or.jp (17 May 2021, date last accessed)). As a result, the numbers of genes classified into HC, LC, and TE were 29,276, 20,645, and 2,754, respectively (Supplementary Table S7).

### 3.4. Gene comparison among the other plant species

The status of the genes in spinach lines (03-009, Sp75 and Viroflay) and the two goosefoot family species (*C. quinoa* and *B. vulgaris*) is summarized in Supplementary Table S8. The HC genes of SOL_r1.1a were found to have high completeness, with a BUSCO score of 94.7%, which is substantially equivalent to those of CDS sequences from the other spinach lines, Sp75 (v1) and Viroflay (SpiSet-1), as well as *C. quinoa* (v1.0) and *B. vulgaris* (BeetSet-2). In contrast, the LC and TE genes of SOL_r1.1a showed extremely low BUSCO completeness scores of 0.4% and 0.1%, respectively. These results suggest that the intrinsic genes were correctly selected as HC genes from the ‘best’ genes predicted by BRAKER2. The genes related to TEs were also selected from the ‘best’ genes. The remaining genes were classified as LC genes, and most of them were unlikely to be intrinsic to spinach; nonetheless, the LC genes can be an informative sequence set to search for novel functional genes, particularly when combined with the expression evidence obtained from future RNA-seq studies of as-yet-unexamined organs/tissues.

The sequence similarities of the genes from the three spinach lines and the two other goosefoot family species were compared by OrthoFinder v2.1.2. The distribution of the genes among the three species is summarized by Venn diagram (Supplementary Fig. S2). The 12,998 orthogroups were commonly distributed among the three species. In the orthogroups, 16,976, 16,175, 16,490, 26,255, and 16,828 genes were respectively included in 03-009, Sp75, and Viroflay in *S. oleracea*, *C. quinoa*, and *B. vulgaris*. There were 11, 3, 3, 31, and 25 orthogroups respectively found in 03-009, Sp75, Viroflay, *C. quinoa*, and *B. vulgaris*, and these orthogroups respectively included 87, 12, 6, 274, and 180 genes. The numbers of genes having no hits against the other plant species among them were 3,977, 3,149, 2,024, 11,475, and 8,098.

### 3.5. Inference of RGAs

By using RGAugury software, the annotated genes in the three spinach lines, 03-009 (HC genes of SOL_r1.1a), Sp75 (v1) and Viroflay (SpiSet-1), as well as *C. quinoa* (v1.0) and *B. vulgaris* (BeetSet-2), were searched for the RGAs. As shown in Supplementary Table S9, the identified RGAs were classified into four groups (NBS, RLP, RLK, and TM-CC) based on their domains, and the NBS-containing proteins were further classified into subgroups based on the domain combinations as follows: CN (CC-NBS), CNL (CC-NBS-LRR), TN (TIR-NBS), TNL (TIR-NBS-LRR), NL (NBS-LRR), TX (TIR-unknown domain), and OTHER (CC-TIR). Of the annotated genes in the three spinach lines, 03-009, Sp75, and Viroflay, and *C. quinoa* and *B. vulgaris*, 695, 751, 557, 1,504, and 732 were identified as RGAs, respectively. Among the three species, *C. quinoa* was shown to have the most RGAs, which is likely due to the polyploidy (2*n* = 4*x* = 36) of this species.^43^ Nearly the same number of RGAs was detected in *B. vulgaris* and the spinach lines except for Viroflay. The lower number in Viroflay (SpiSet-1) was probably due to the lower coverage of the genome assembly.

Among the 695 RGAs in 03-009 (SOL_r1.1a), RLKs accounted for the largest number, i.e. 416 (59.8% of all RGAs), followed by NBS-containing proteins with 114 (16.4%), TM-CC-containing proteins with 95 (13.7%) and RLPs with 70 (10.0%; Supplementary Table S9). In the other spinach lines, as well as in *C. quinoa* and *B. vulgaris*, RLKs accounted for the highest proportion of the detected RGAs. These results agree with the finding that the prevalence of RLKs is commonly observed among angiosperm genomes (Table 2 in 39) In the NBS-containing proteins of SOL_r1.1a, CC-NBS was shown to be the most common domain arrangement (42 out of 114 proteins), followed by NBS-LRR (38 out of 114), and a similar distribution was apparent in the NBS-containing proteins of the other spinach lines, *C. quinoa* and *B. vulgaris* (Supplementary Table S9). The larger proportions of CC-NBS and NBS-LRR members were also found in NBS-containing proteins identified in the EL10 *B. vulgaris* genome assembly.^53^ Considering the above, it may be reasonable to conclude that the sets of RGAs identified in this study were properly predicted and will be informative for identifying novel disease-resistance genes.

### 3.6. Construction of a high-density linkage map and pseudomolecules, representing the spinach chromosomes

**Figure 1:**
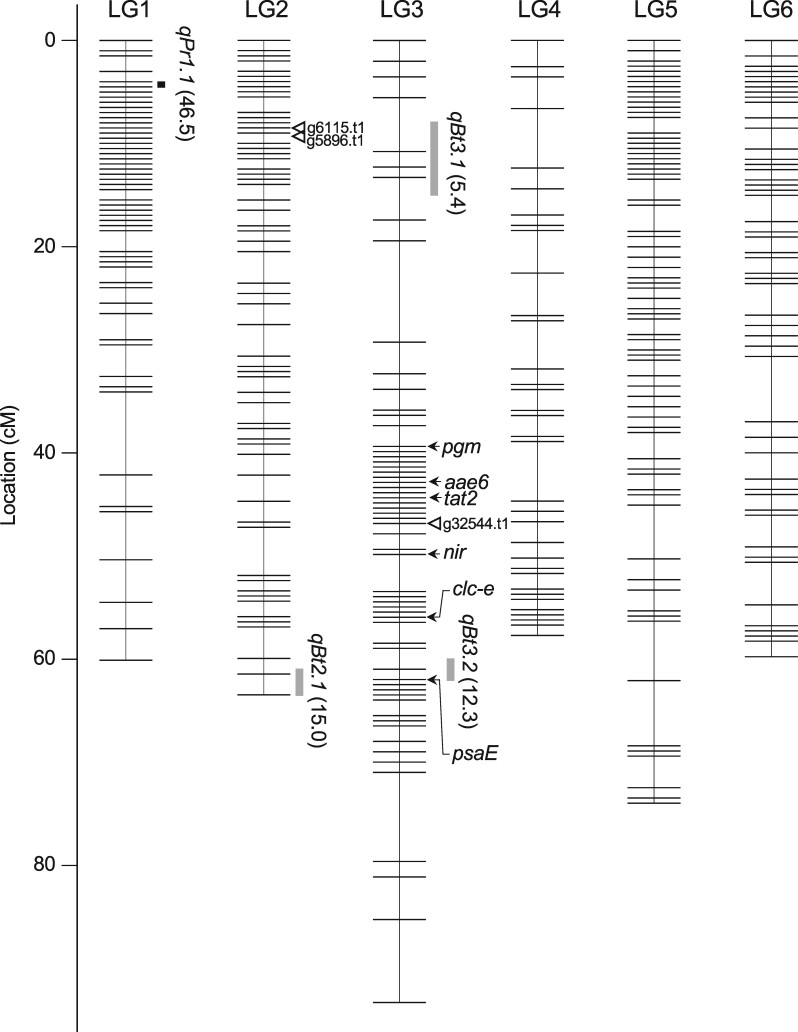
Molecular linkage map of spinach, based on the mapping population, 03-009 × 03-336 F_2_. Black and grey bars on the right side of the map represent QTLs for fruit/seed shape and bolting time, respectively, whose LOD values are given in parentheses. Arrows indicate the loci of protein-coding genes, which were mapped on the sex chromosomes in our previous study.^12^ Open triangles indicate the loci of Sol_r1.0_p041.1.g32544.t1, Sol_r1.0_p003.1.g5896.t1, and SoCOL1, with significant homology to *BTC1*, *BvBBX19* and *CONSTANTS*, respectively.

DNA samples of F_1_ and F_2_ plants from the cross 03-009 × 03-336 and their parental plants were subjected to ddRAD-seq analysis, which yielded reads from each plant ranging from 3,908,570 to 13,051,264 (average: 7,652,625) (Supplementary Table S3). The ddRAD-seq reads were mapped to the spinach genome assembly SOL_r1.1, and a total of 156,455 single nucleotide polymorphism (SNP) candidates were obtained. 1,780 and 2,206 SNP sites were found to be compatible with the “stringent” and ‘less stringent’ quality/depth-criteria combined with the genotype criterion (see Materials and methods), respectively. Moreover, 882 of the 2,206 ‘less stringent’ sites were extracted to be arranged at ∼0.8 Mb intervals on scaffolds of SOL_r1.1, and then used for the following analysis together with the 1,780 ‘stringent’ SNP sites (in total, 2,247 SNP sites remained after excluding 415 redundant sites). Among the 2,247 SNP loci, 2,176 showed the expected 1:2:1 segregation ratio [*χ*^2^-test, *P* (without Bonferroni correction) > 0.001] in the F_2_ progeny. The linkage analysis on the F_2_ population arranged the 2,176 SNPs into the expected number (*n *=* *6) of linkage groups with a number of SNP markers ranging from 265 to 460, which were designated LG1–LG6 in descending order by the number of SNPs included. The sizes of the linkage groups range from 57.7 cM (LG4) to 93.2 cM (LG3), totalling 411.1 cM (Fig. 1 and Table 1), and the largest gap, 9.8 cM, was found on the linkage group LG3.

**Table 1:** Summary of the genetic linkage map and the pseudomolecules for spinach

Linkage groups	Genetic length (cM)	Number of SNPs	Pseudomolecules	Physical length (bp)
LG1	60.5	460	SOL_r1.0_chr01	146,198,663
LG2	64.6	433	SOL_r1.0_chr02	124,699,619
LG3	94.7	390	SOL_r1.0_chr03	133,520,741
LG4	57.7	347	SOL_r1.0_chr04	109,917,612
LG5	73.9	281	SOL_r1.0_chr05	88,246,263
LG6	59.7	265	SOL_r1.0_chr06	85,406,467
Total	411.1	2176	—	687,989,365

**Figure 2:**
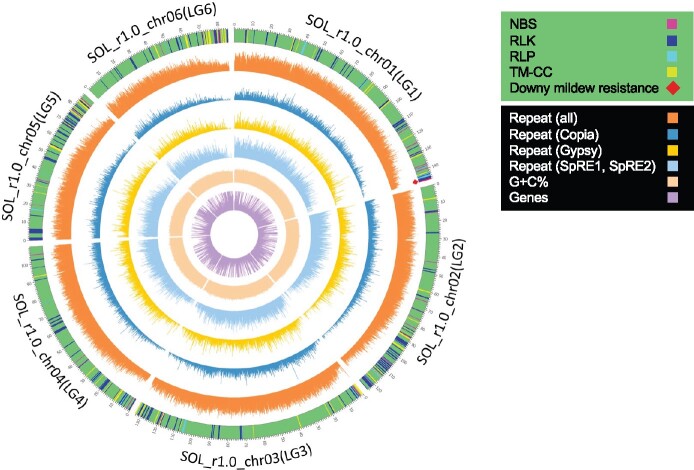
Genome map of the SOL_r1.0_pseudomolecule. Identifiers of the six pseudomolecules and their associated linkage groups are displayed around the genome map. The locations of the RGAs, NBS-encoding proteins, RLKs, RLPs, and TM-CC proteins, are indicated on the outermost genome map. Red diamonds on the outermost circular map indicate the locations of the downy mildew resistance genes corresponding to Spo12729 (Sol_r1.0_p006.1.g25710.t1), Spo12784 (Sol_r1.0_p006.1.g25727.t7), and Spo12903 (Sol_r1.0_p006.1.g25767.t1). The inner circular maps show percentage of the total repeat sequences (the repeat sequences classified into *Copia*, *Gypsy*, *SpRE1*, and *SpRE2*), each of the repeat sequence and GC contents, and the number of genes calculated by using a window size of 50 kb with a sliding window size of 10 kb.

The linkage map comprising the 2,176 SNPs anchored 112 scaffolds of SOL_r1.1 (Supplementary Table S10), the total length of which was 913.3 Mb. Although 83 of the 112 scaffolds were respectively anchored to a region on a single linkage group, each of the remaining 29 scaffolds was assigned to multiple regions from different linkage groups, being judged to be chimeric, presumably resulting from scaffolding errors. Taking these results into account, six pseudomolecules representing spinach chromosomes were constructed as follows: a total of 135 sequence segments with individual lengths of >1.0 kb (a total combined length of 686.6 Mb) (Supplementary Table S10), which were spanned by the SNPs anchoring the scaffolds, were oriented and ordered with reference to the linkage map, and were catenated while filling in the gaps between them with Ns of 10 kb in length. The resultant pseudomolecules were made publicly available in Spinach Genome DataBase (http://spinach.kazusa.or.jp (17 May 2021, date last accessed)), and named SOL_r1.0_pseudomolecule (chr01 to chr06), which are associated with linkage groups LG1 to LG6, respectively, and ranged from 85,406,467 (SOL_r1.0_chr06) to 146,198,663 bp (SOL_r1.0_chr01) in length, with a total length of 687,989,365 bp (Table 1 and Fig. 2) and a BUSCO completeness of 82.3%. The status of the pseudomolecules for 03-009 (SOL_r1.0_pseudomolecule) and Sp75 is also shown in Supplementary Table S11. Length and BUSCO score of SOL_r1.0_pseudomolecule were much improved compared to those of the previously published pseudomolecules in Sp75 (spinach_genome_v1 Sochrs1–6: total length 463,559,193 bp, BUSCO complete = 70.4%) (ftp://spinachbase.org/pub/spinach/genome/spinach_genome_v1.fa.gz; Supplementary Table S11). Furthermore, the SOL_r1.0_pseudomolecule also has a much higher LAI score (16.1) than that of the pseudomolecules in Sp75 v1.0 (LAI = 4.5). Consistent with these findings, RepeatMasker identified a higher proportion of repetitive sequences in the SOL_r1.0_pseudomolecule than in the pseudomolecules of Sp75 v1.0: repetitive sequences were found to account for 72.0% and 47.4% of the former and the latter pseudomolecules, respectively (Supplementary Table S6 and Fig. 2).

The HC genes predicted in SOL_r1.1a were mapped against the pseudomolecule (SOL_r1.0_pseudomolcule) by GMAP v2020.06.01^54^ (Fig. 2). A total 17,869 of the 29,276 (61.0%) genes were mapped, and those with length coverages of 95% to 105% and sense direction of splicing site were selected. Among the genes mapped to the pseudomolecule, 396 were found to be RGAs: 60 NBS domains, 244 RLKs, 31 RLPs and 61 TM-CCs (Supplementary Table S9). In recent studies, the spinach downy mildew resistance gene, *RPF1*, was mapped to a distal region (position 0.34–1.23 Mb) on a pseudomolecule, Sochr3, in the previous spinach genome assembly, spinach_genome_v1.^4^^,^^25^ As shown in Fig. 2, the *RPF1* candidates (Spo12729, Spo12784, and Spo12903) encoding the RLP and NBS-LRR domains^25^ were mapped to a distal region (physical position, 145.7–146.1 Mb) of SOL_r1.0_chr01, suggesting that the chromosomal region is likely to correspond to the *RPF1* locus. The three *RPF1* candidates, Spo12729, Spo12784, and Spo12903, were likely to correspond to Sol_r1.0_p006.1.g25710.t1, Sol_r1.0_p006.1.g25727.t7, and Sol_r1.0_p006.1.g25767.t1 located at the distal region of SOL_r1.0_chr01 (Supplementary Table S9).

The 3 Mb region surrounding the potential *RPF1* locus on SOL_r1.0_chr01 (143–146 Mb) was found to carry five NBS domains, three RLPs and four RLKs, respectively (Supplementary Table S9). As shown in Supplementary Fig. S3, a phylogenetic analysis of the NBS domains mapped on the pseudomolecules showed that the five NBS domain genes formed a clade with the *RPF1* candidate (Spo12784 and Spo12903).^4^^,^^25^ Although the clade included NBS domains mapped to not only chr01 but also chr02, chr03 and chr05, the basal gene (Sol_r1.0_p006.1.g26028.t6) was located at the distal chr01. This may suggest that local duplication events play an important role in the evolution of the downy mildew resistance in spinach, as in the case of the generation of rhizomania resistance of sugar beet.^53^ However, a phylogenetic analysis did not support a significant contribution of local duplication events in the generation of the RLP gene (Spo12729) reported as the *RPF1* candidate (data not shown). Further analysis is needed to confirm this hypothesis.

It is worth noting that the longest linkage group (LG3) and the pseudomolecule corresponding thereto (SOL_r1.1_chr03) were found to represent the sex chromosomes, as the protein coding genes (*phosphoglucomutase* (*pgm*)/Sol_r1.0_p001.1.g22838.t1, acyl-activating enzyme 6 (*aae6*)/Sol_r1.0_p002.1.g17189.t1, aminotransferase (*tat2*)/Sol_r1.0_p006.1.g27450.t3, *nitrite reductase* (*nir*)/Sol_r1.0_p061.1.g9296.t1, *chloride channel protein* (*clc-e*)/Sol_r1.0_p062.1.g49095.t1, photosystem I subunit IV (*psaE*)/Sol_r1.0_p055.1.g31390.t1), which were mapped on the sex chromosomes in our previous study,^12^ are located on LG3/SOL_r1.0_chr03 in the proper order (Fig. 1). In the previous spinach genome assembly (spinach_genome_v1), a pseudomolecule, Sochr4, was considered likely to represent the sex chromosomes.^4^^,^^12^^,^^55^ Furthermore, the male determining locus was reported to be located between the *aae6* and *tat2* genes, and the locus for monoecism was reported to be closely linked to *clc-e*.^12^^,^^20^^,^^27^ This may provide a useful basis for isolating the sex-determining genes and for developing sex-diagnostic markers suitable for female and male parents in a hybrid seed production system.^27^

### 3.7. Mapping of QTLs for bolting timing and fruit/seed shape

As shown in Supplementary Fig. S4, line 03-009 (*n* = 7) and line 03-336 (*n* = 8) bolted on average 6.3 (ranging from 5 to 8) and 18.4 (14–23) days after starting long-day treatment, respectively, and these bolting times were significantly different (Exact Wilcoxon rank sum test, *W *=* *0, *P *<* *0.001). The F_1_ progeny (*n* = 7) from these lines showed an intermediate bolting time (10–11 days) compared with the parental lines. In the 03-009 × 03-336 F_2_ population (*n* = 101), the days to bolting from the start of long-day treatment varied from 7 to 34 (on average 15.3 days). As illustrated in Supplementary Fig. S5, lines 03-009 and 03-336 bore prickly and round seeds, respectively, and the F_1_ progeny from the lines produced prickly seeds. Among the 101 F_2_ individuals, 19 either died before seed set, or exhibited complete or near-complete failure to set seeds due to the highly-male monoecious conditions (plants having little or no female and hermaphrodite flowers). The remaining 82 F_2_ individuals consisted of 60 prickly seeded and 22 round-seeded plants, which fit the Mendelian ratio of 3:1, and was consistent with the findings of Nohara^22^ (see Introduction). The two phenotype data sets for the F_2_ population were used for QTL analyses with the linkage map constructed in this study.

The QTL analysis of the bolting time identified three significant QTLs, designated *qBt2.1* (LOD = 15.0) on LG2, and *qBt3.1* (LOD = 5.4) and *qBt3.2* (LOD = 12.3) on LG3. The three QTLs accounted for 59.2% of the total phenotypic variance explained (PVE): *qBt2.1* exhibited the highest PVE value (31.7%) among them, followed by *qBt3.2* (20.0%) and *qBt3.1* (7.5%) (Fig. 1 and Supplementary Figs S6–8). The locus for the prickle development was mapped to a distal region on LG1, and was designated *qPr1.1*, which was detected with a high LOD value of 46.5 and accounted for 88.1% of the phenotypic variance (Fig. 1 and Supplementary Fig. S9).

### 3.8. Candidate genes underlying the QTLs for bolting time

To search for the causal genes underlying the QTLs (*qBt2.1*, *qBt3.*1, and *qBt3.2*) for bolting time, we referred to the *Arabidopsis* flowering genes listed in the file (flowering_time_genes.xls) provided by the Department of Plant Developmental Biology at the Max-Planck Institute (https://www.mpipz.mpg.de/25162/flowering_time_genes.xls (17 May 2021, date last accessed)). As illustrated in Supplementary Fig. S7, the SNPs associated with *qBt3.1* are located on the end of scaffold Sol_r1.0_p065.1 (approximately position 4–5 Mb), and the SNPs within *qBt3.2* are located across the ends of scaffolds p055.1 and p096.1 (Supplementary Fig. S8). As shown in Supplementary Figs S7–8 and Supplementary Table S12, the genomic regions corresponding to *qBt3.1* and *qBt3.2* were found to carry three and two homologues of *ArabidopsisFT*,^56^^,^^57^ respectively. No other *FT* homolog was found in SOL_r1.1. As shown in Supplementary Fig. S10, based on phylogenetic analysis, the five spinach *FT* homologues were classified into three groups. The first group consists of Sol_r1.0_p096.1.g1617.t4 and Sol_r1.0_ p055.1.g31399.t1 located in *qBt3.2*, which was designated *SoFT1a* and *SoFT1b*, respectively. The second one is composed of two homologues (Sol_r1.0_p065.1.g24569.t1 and Sol_r1.0_p065.1.g24571.t1, designated *SoFT2a* and *SoFT2b*, respectively) located in *qBt3.1*. Sugar beet has two *FT* homologues, *BvFT1* and *BvFT2*, which are a pair of antagonistic regulators in the flowering pathway: *BvFT2* is an orthologue of *Arabidopsis FT*, and functions as a floral activator, whereas *BvFT1* is a repressor of flowering.^58^*SoFT2a* and *SoFT2b* formed a clade (supported by a bootstrap value of 76%) along with sugar beet *BvFT2*. The other homolog, Sol_r1.0_p065.1.g24573.t2 located in *qBt3.1*, was classified into the third group and named *SoFT3*. Although only *SoFT2a* and *SoFT2b* were clustered together with *BvFT2*, with a high bootstrap value, the other spinach *FT* homologues are unlikely to be floral repressors, since the five spinach *FT* homologues contain amino acid residues identical to those of *BvFT2*, which are critical for the antagonistic activity (data not shown). This result is consistent with the previous report that orthologues of *BvFT1* are likely to be restricted to the genus *Beta.*^58^ In addition to the *FT* homologues, the QTL region of *qBt3.2* was also found to harbour a gene (Sol_r1.0_p055.1.g31358.t2) related to *Arabidopsis AGL24* (*AGAMOUS-like 24*) and *AGL22* (*AGAMOUS-like 22*)/*SVP* (*SHORT VEGETATIVE PHASE*) (Supplementary Table S12 and Fig. S8). *AGL24* is a promoter of flowering, whereas *AGL22* (*AGAMOUS-like 22*)/*SVP* is a floral repressor.^59^

As shown in Supplementary Fig. S6, *qBt2.1* corresponds to scaffold Sol_r1.0_p057.1 (approximately position 1.25–2.70 Mb) in SOL_r1.1. This region contains a gene (Sol_r1.0_ p057.1.g23639.t1) with homology to *Arabidopsis FLC* and its paralogues (e.g. *MADS AFFECTING FLOWERING 4/AGAMOUS LIKE 69*), as well as the sugar beet *FLC* homolog, *FLC-like 1* (*BvFL1;*Supplementary Table S12 and Fig. S11). Given that, as in sugar beet, the spinach *FLC* homolog does not play a major role in the vernalization pathway,^17^ Sol_r1.0_ p057.1.g23639.t1 may need to be excluded from the candidates for *qBt2.1*. Moreover, since the frequency distribution of bolting time in the F_2_ population suggested that the 03-009 allele at *qBt2.1* acted dominantly to result in early flowering under long-day conditions (Supplementary Fig. S12), it may be reasonable to assume that the QTL region contains a floral promoter rather than a repressor functionally similar to *Arabidopsis FLC*. In sugar beet, *BTC1* and *BvBBX19* were isolated as major genetic components of the photoperiod‐dependent flowering pathway, acting upstream of *BvFT1* and *BvFT2*. *BTC1* and *BvBBX19* share homology to *Arabidopsis CONSTANS* (*CO*), but unlike *CO*, the sugar beet genes encode proteins lacking either a B-Box or a CCT domain.^18^^,^^19^^,^^60^ Spinach genes, Sol_r1.0_p003.1.g5896.t1, Sol_r1.0_p041.1.g32544.t1, and Sol_r1.0_p003.1.g6115.t1 (*SoCOL1*), with significant homology to *BTC1*, *BvBBX19*, and *CO* (blast *e*-value = 0.0, 1.36E-80 and 5e-56, respectively) (Supplementary Figs S13 and S14), were not found in *qBt2.1*, and were also not found in the other two QTLs for bolting time (Fig. 1). This makes it likely that a novel floral promoter underlies *qBt2.1*, although the direct proof remains to be established.

### 3.9. Candidate genes within a genomic region, *qPr1.1*, associated with prickle development

As shown in Supplementary Fig. S9, the QTL, *qPr1.1*, for the prickle development was located to a region on scaffold Sol_r1.0_p007.1. The 95% CI of *qPr1.1* is bracketed by two SNP markers spaced by a small physical distance of 1.37 Mb. To identify the candidate genes for *qPr1.1*, the QTL region was searched for homologues of the genes associated with development of not only prickles, but also of awns in cereals, warts and trichomes, since prickles are defined as an outgrowth of epidermal tissues. In the 1.37 Mb region, genes (Sol_r1.0_p007.1.g35658.t1 and Sol_r1.0_p007.1.g35660.t1) with homology to the salicylic acid-binding protein 2-like gene and methyl esterase 1 gene were found, which are associated with the jasmonic acid metabolic (GO:0080032) and salicylic metabolic (GO:0080031) processes (Supplementary Table S12).^61^ Another gene, Sol_r1.0_p007.1.g35699.t1, located in this region also has similarity to a gene (*ATST2A*, AT5G07010) involved in the jasmonic acid metabolic process^62^ (Supplementary Fig. S9 and Table S12). Jasmonic acid has been reported to play significant roles in trichome development.^63^ Furthermore, the *EPIDERMAL PATTERNING FACTOR-like* (*EPFL*) protein gene (Sol_r1.0_p007.1.g35742.t1) was found to be located close (401.5 kb) to the 1.37 Mb region corresponding to the 95% CI of *qPr1.1* (Supplementary Fig. S9 and Table S12). In rice, a regulator of awn development, *RAE2*, was shown to encode an EPFL protein.^64^ Members (Sol_r1.0_p007.1.g35769.t1 and Sol_r1.0_p007.1.g35771.t1) of the C2H2 zinc finger protein (ZFP) gene family and R2R3-MYB gene family were also found near this QTL region (Supplementary Fig. S9 and Table S12). Certain C2H2 ZFP and R2R3-MYB genes were shown to be involved in the development of prickles, awns, warts, and trichomes.^63^^,^^65–67^

In this study, the use of SOL_r1.1 coupled with the ddRAD-seq technique was shown to facilitate quick identification of the QTLs for the agronomic traits. In this context, SOL_r1.1 could be useful as a reference genome sequence for future QTL-seq analysis and genome-wide association studies to identify novel loci important in spinach breeding programs. The SNPs underlying the QTLs identified here may be valuable for the development of molecular markers to select for the traits in question, and further analysis of the identified candidate genes may help elucidate the molecular mechanisms regulating bolting time and fruit/seed shape in spinach.

## Supplementary data

Supplementary data are available at DNARES online.

## Supplementary Material

dsab004_Supplementary_Data
