# Supplementary material for: A spinach genome assembly with remarkable completeness, and its use for rapid identification of candidate genes for agronomic traits
Source: DNA Res. 2021 Jun 17;28(3):dsab004. doi: 10.1093/dnares/dsab004 (PMC8231376; doi:10.1093/dnares/dsab004)
Supplement: dsab004_Supplementary_Data [file dsab004_supplementary_data.zip › SupFigs_S1_S14.pdf]

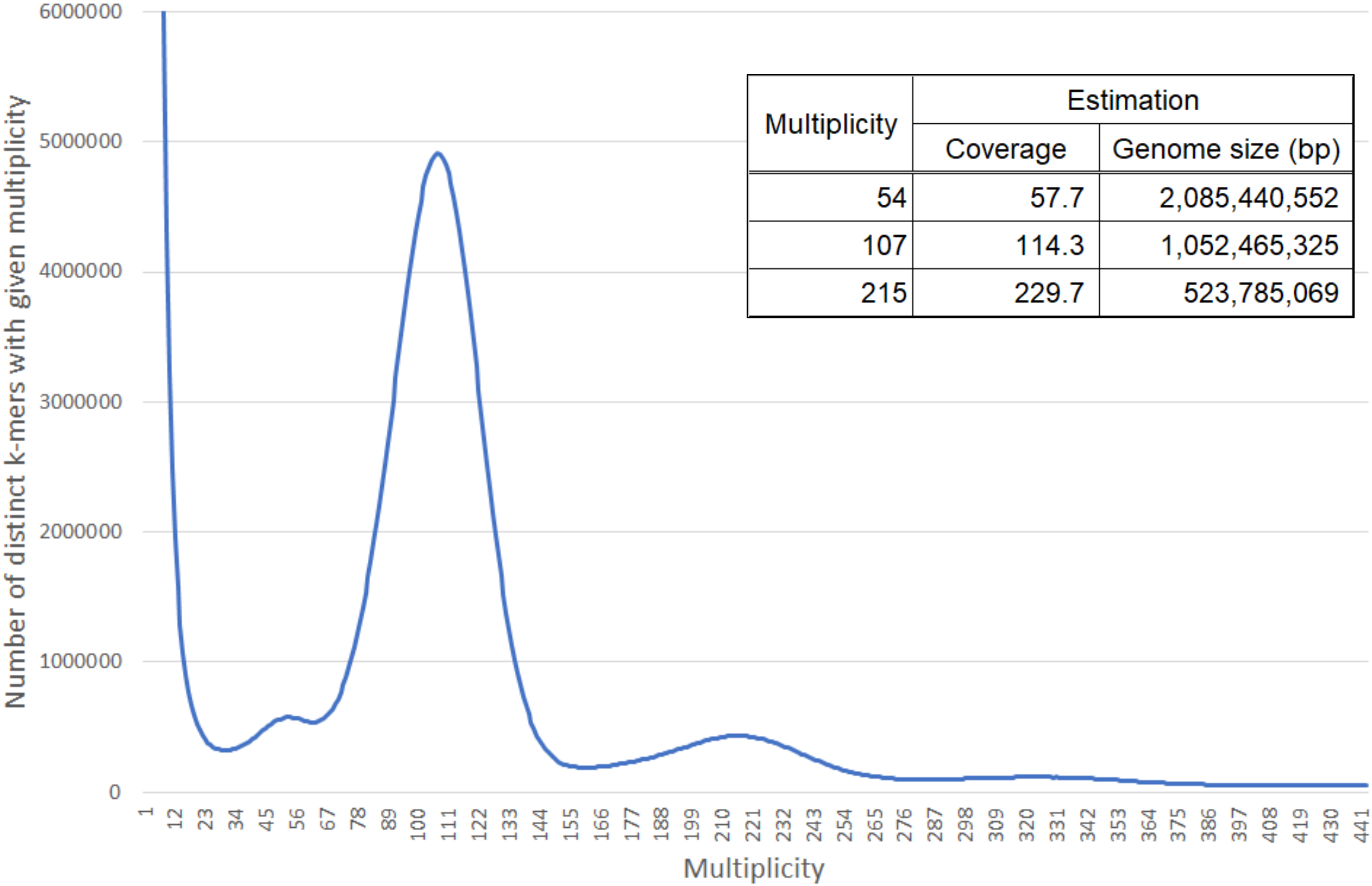

Multiplicity	Estimation	
	Coverage	Genome size (bp)
54	57.7	2,085,440,552
107	114.3	1,052,465,325
215	229.7	523,785,069

Supplementary Figure S1. Hirakawa et al.

Supplementary Figure S1. Kmer frequency plot of 03-009

The Illumina paired-end reads (DRX241308) were used to calculate the kmer frequency. The kmer size used was 17. According to the peak at multiplicity of 107, the genome size was estimated to be 1.05 Gb.

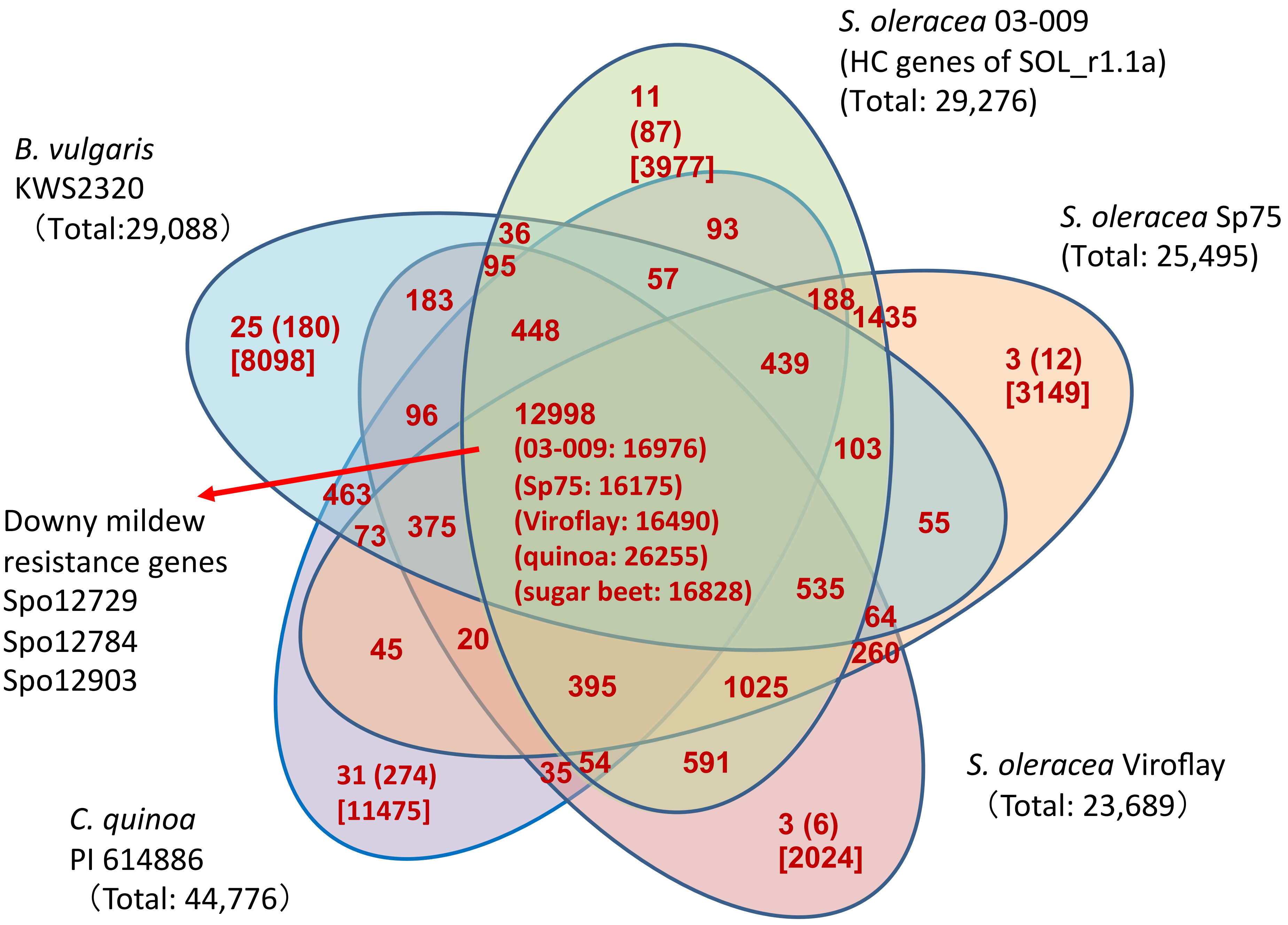

Supplementary Figure S2. Hirakawa et al.

Supplementary Figure S2. Gene comparison among the three species

The genes of the spinaches 03-009 (HC of SOL_r1.1a), Sp75 (v1.0), and Viroflay (SpiSet-1), quinoa (v1) and sugar beet (BeetSet-2) were compared by OrthoFinder. The numbers of orthogroups are shown in each intersection. The numbers of genes classified into the orthogroups are shown in parentheses. The numbers of genes that were not assigned to orthogroups are shown in square brackets. The downy mildew genes corresponding to Spo12729 (Sol_r1.0_p006.1.g25710.t1), Spo12784 (Sol_r1.0_p006.1.g25727.t7), and Spo12903 (Sol_r1.0_p006.1.g25767.t1) were located on the intersection that was common to the five species.

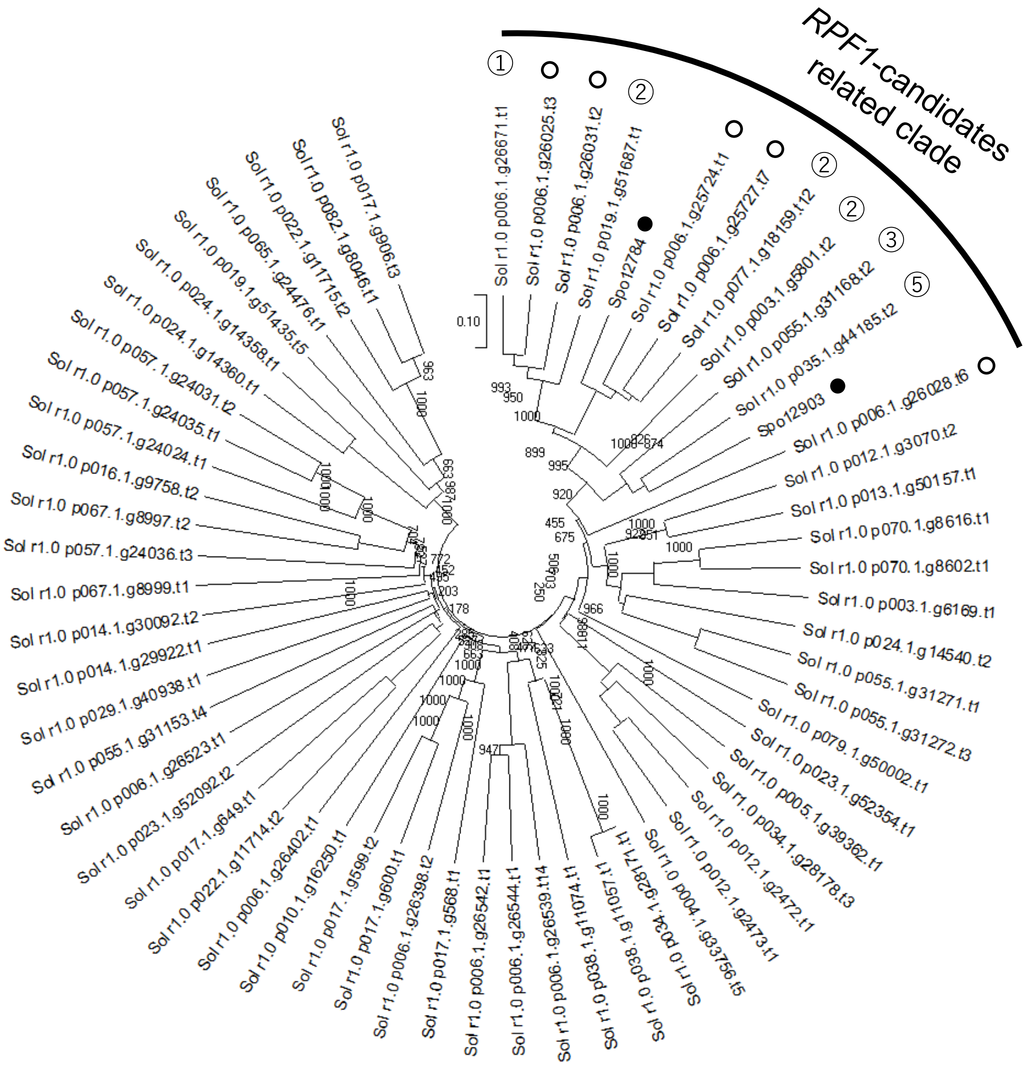

Supplementary Figure S3. Neighbor-joining phylogenetic tree of nucleotide-binding site (NBS) domain proteins encoded in SOL_r1.0_pseudomolecules

The NJ tree is composed of 60 NBS domain proteins encoded in SOL_r1.0_pseudomolecules, and 2 NBS domain proteins (indicated with black dots) encoded by *RPF1*-candidates (Spo12784 and Spo12903; She et al. 2018). NBS domain proteins encoded in the 3 Mb chromosomal region surrounding the *RPF1* locus on Chr01 are indicated with open dots. Within the *RPF1* candidates-related clade, the ID numbers of chromosomes/pseudomolecules from which the NBS domain proteins were derived are indicated by circled numbers.

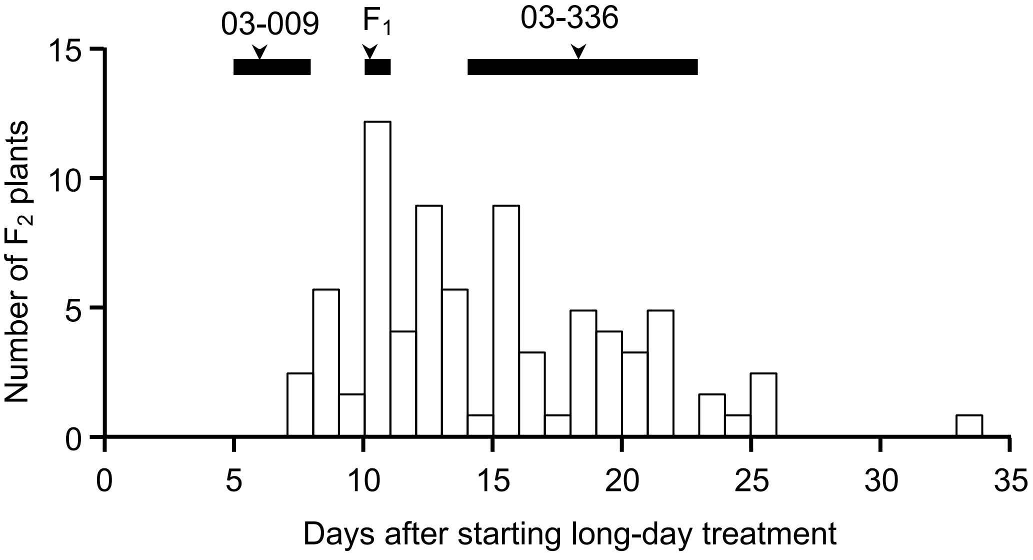

Supplementary Figure S4. Frequency distribution of the bolting time in the 101 F_2 progeny plants from the cross between 03-009 and 03-336 Arrowheads and horizontal bars indicate average numbers and ranges, respectively, of days to bolting of the parental lines, 03-009 and 03-336, and the F_1 progeny.

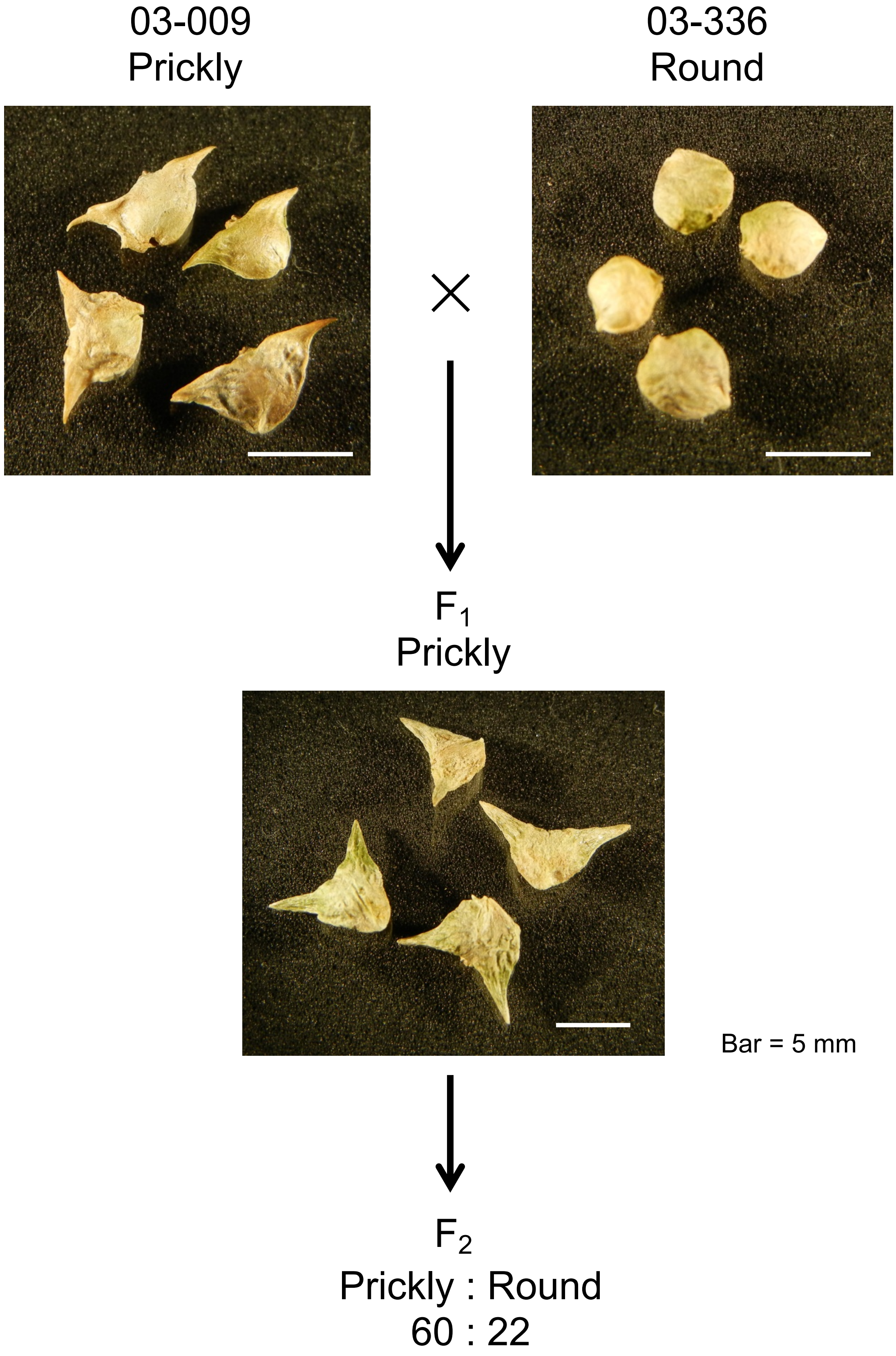

Supplementary Figure S5. Inheritance of fruit/seed shape in spinach

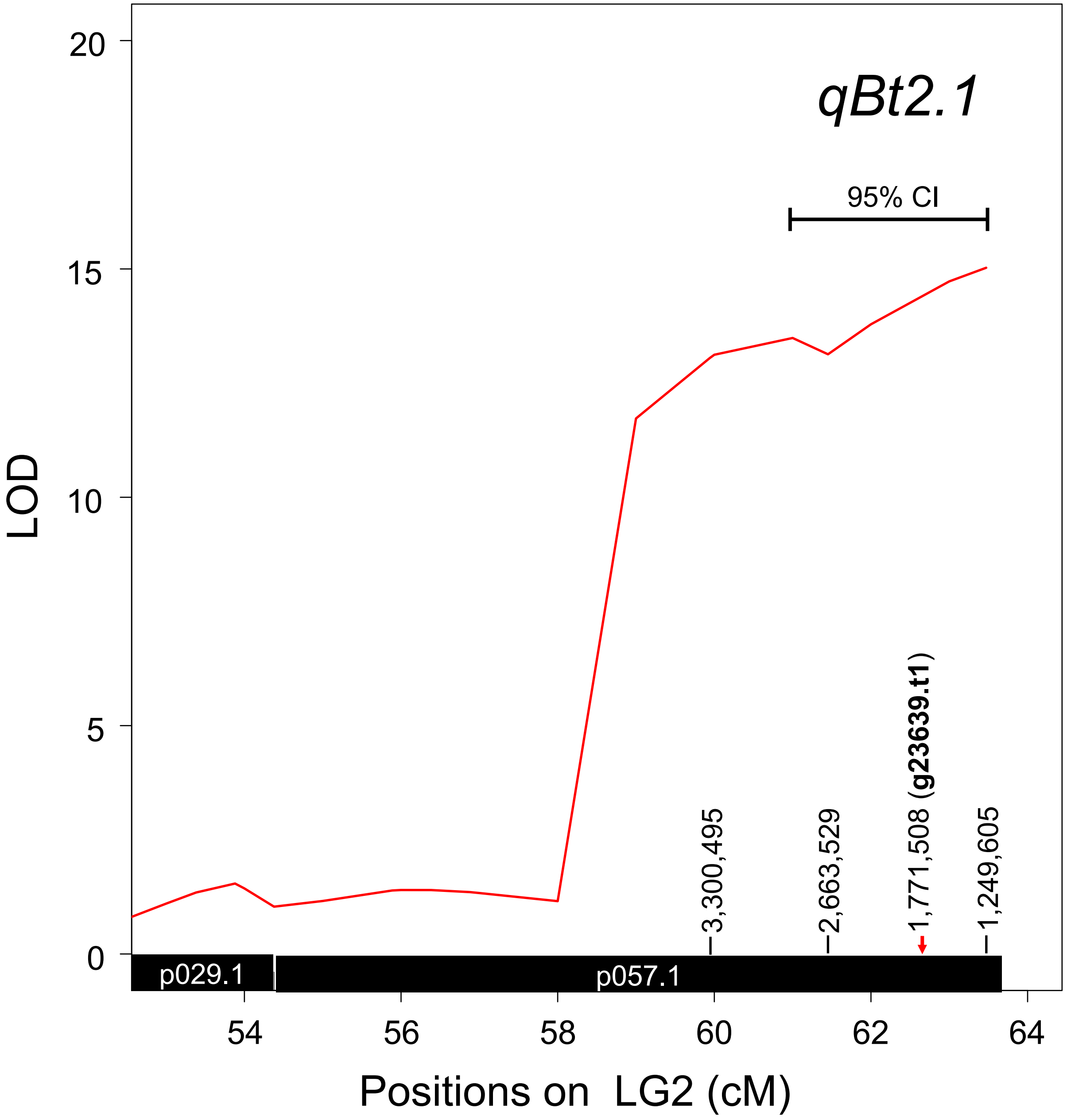

Supplementary Figure S6. A QTL, *qBt2.1*, for bolting timing detected on LG2 Black bars along the horizontal axis represent scaffolds of genome assembly SOL_r1.1 associated with *qBt2.1*. Vertical black lines and red arrows on the scaffolds indicate the nucleotide positions of SNP markers and candidate genes for *qBt2.1*, respectively. The prefix (Sol_r1.0) of the scaffold and the gene ID are omitted.

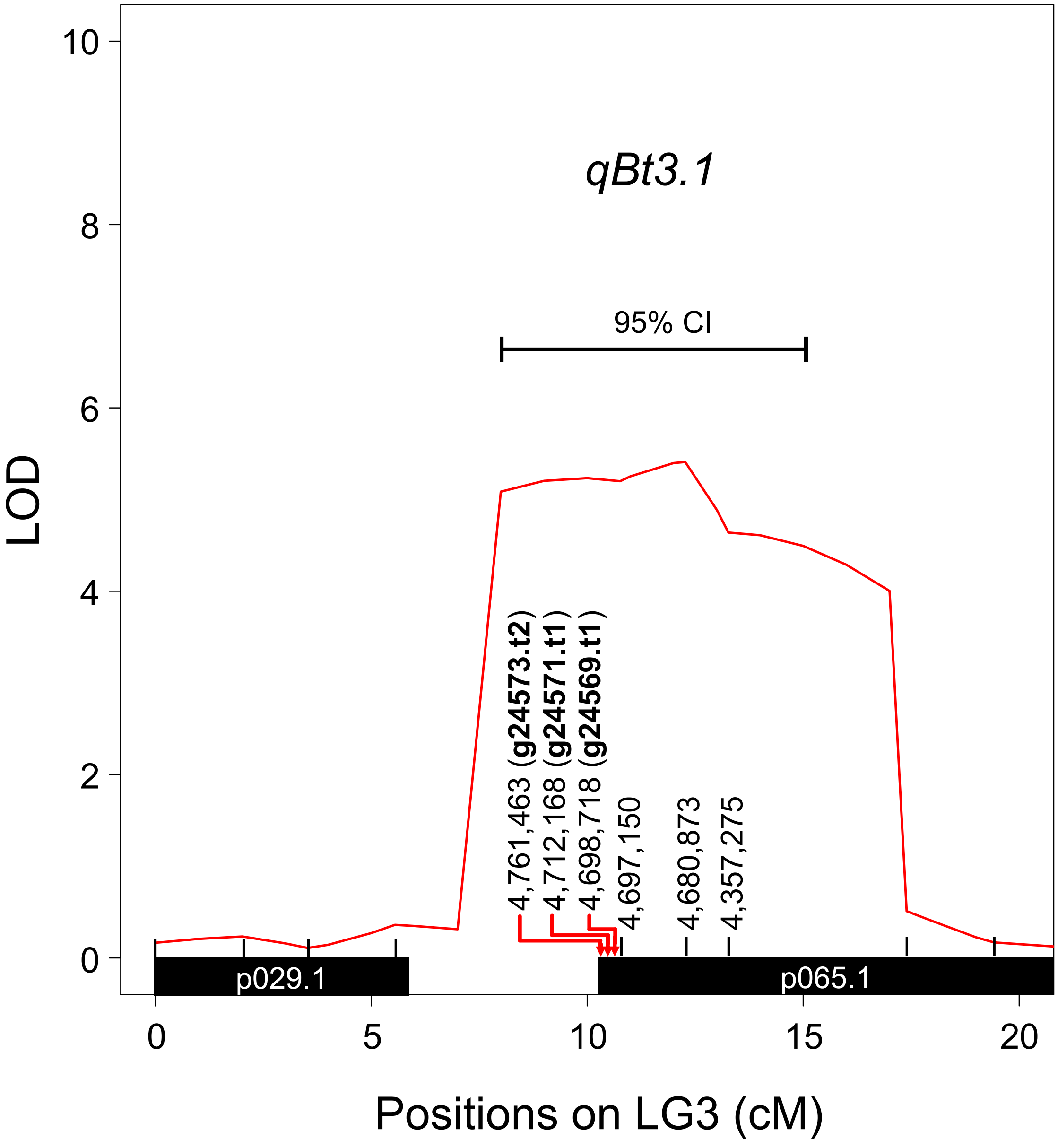

Supplementary Figure S7. A QTL, *qBt3.1*, for bolting timing detected on LG3 Black bars along the horizontal axis represent scaffolds of genome assembly SOL_r1.1 associated with *qBt3.1*. Vertical black lines and red arrows on the scaffolds indicate the nucleotide positions of SNP markers and candidate genes for *qBt3.1*, respectively. The prefix (Sol_r1.0) of the scaffold and the gene ID are omitted.

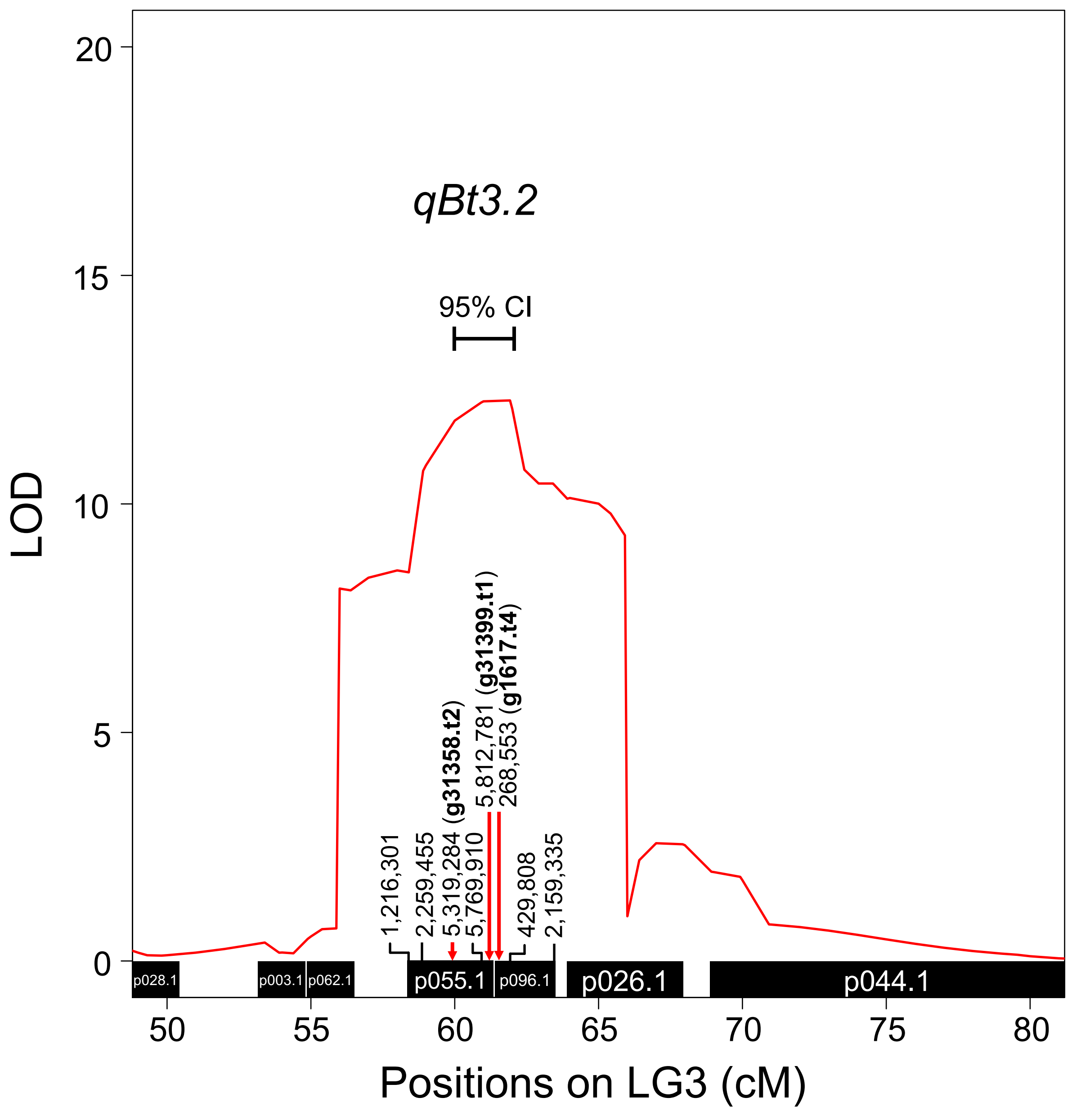

Supplementary Figure S8. A QTL, *qBt3.2*, for bolting timing detected on LG3 Black bars along the horizontal axis represent scaffolds of genome assembly SOL_r1.1 associated with *qBt3.2*. Vertical black lines and red arrows on the scaffolds indicate the nucleotide positions of SNP markers and candidate genes for *qBt3.2*, respectively. The prefix (Sol_r1.0) of the scaffold and the gene ID are omitted.

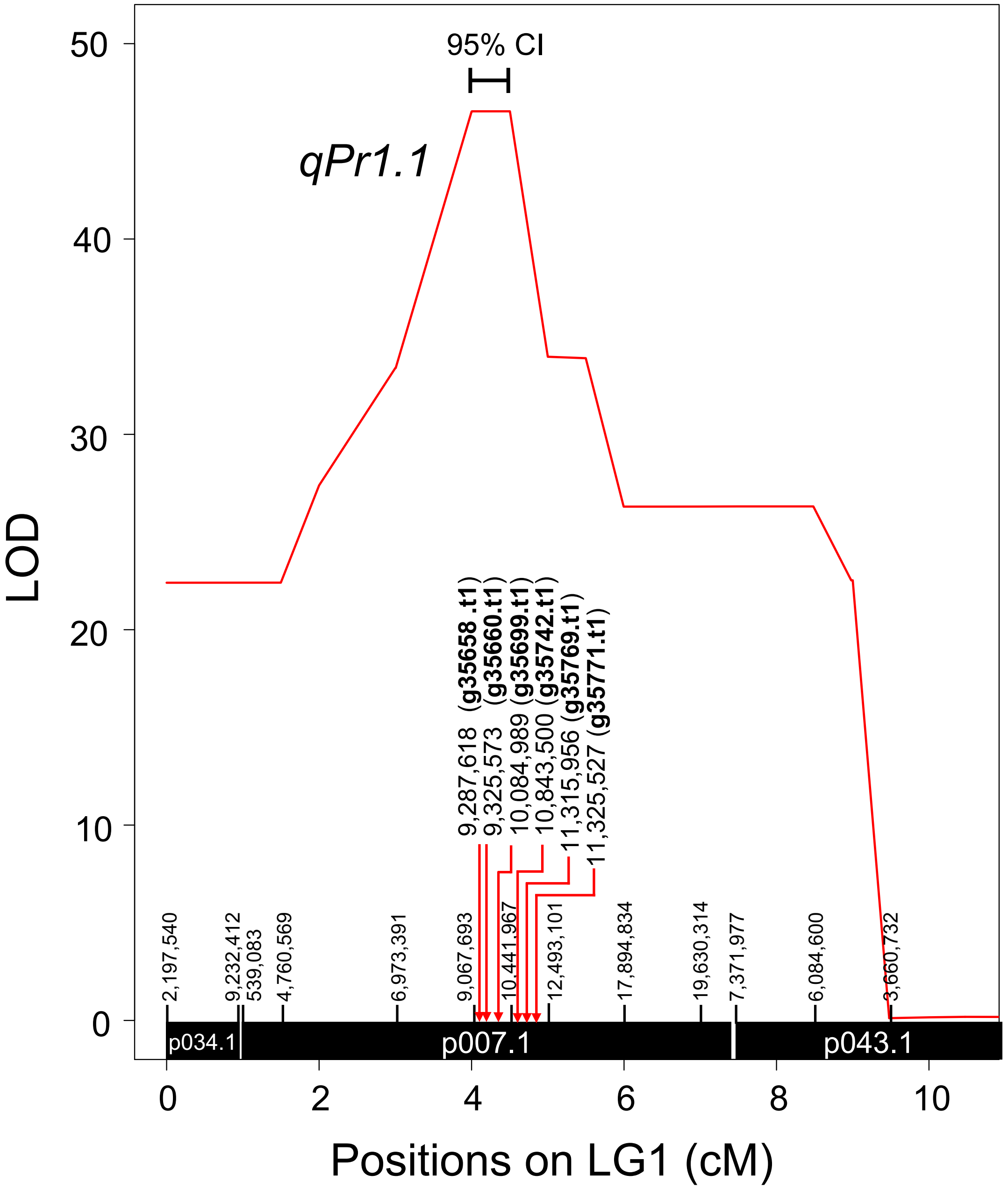

Supplementary Figure S9. A QTL, *qPr1.1*, for fruit/seed shape detected on LG1 Black bars along the horizontal axis represent scaffolds of genome assembly SOL_r1.1 associated with *qPr1.1*. Vertical black lines and red arrows on the scaffolds indicate the nucleotide positions of SNP markers and candidate genes for *qPr1.1*, respectively. The prefix (Sol_r1.0) of the scaffold and the gene ID are omitted.

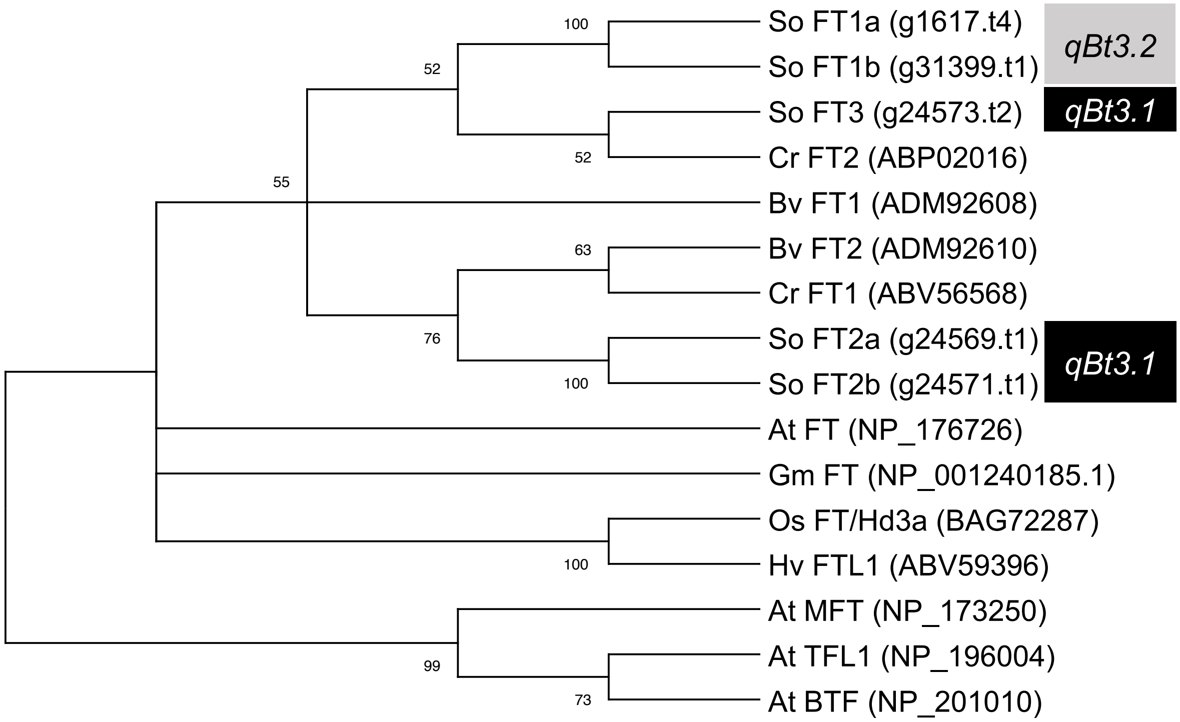

Supplementary Figure S10. Neighbor-joining phylogenetic tree of the amino acid sequences of FT homologs

Arabidopsis phosphatidylethanolamine binding proteins (PEBPs), TFL1, BFT and MFT, were used as outgroups. Plant species are represented by two letters as follows: So, *Spinacia oleracea*; Os, *Oryza sativa*; Bv, *Beta vulgaris*; Gm, *Glycine max*; At, *Arabidopsis thaliana*; Cr, *Chenopodium rubrum*; Hv, *Hordeum vulgare*. The gene IDs (written in abbreviated forms) of SOL_r1.1a and the GenBank IDs are given in parentheses.

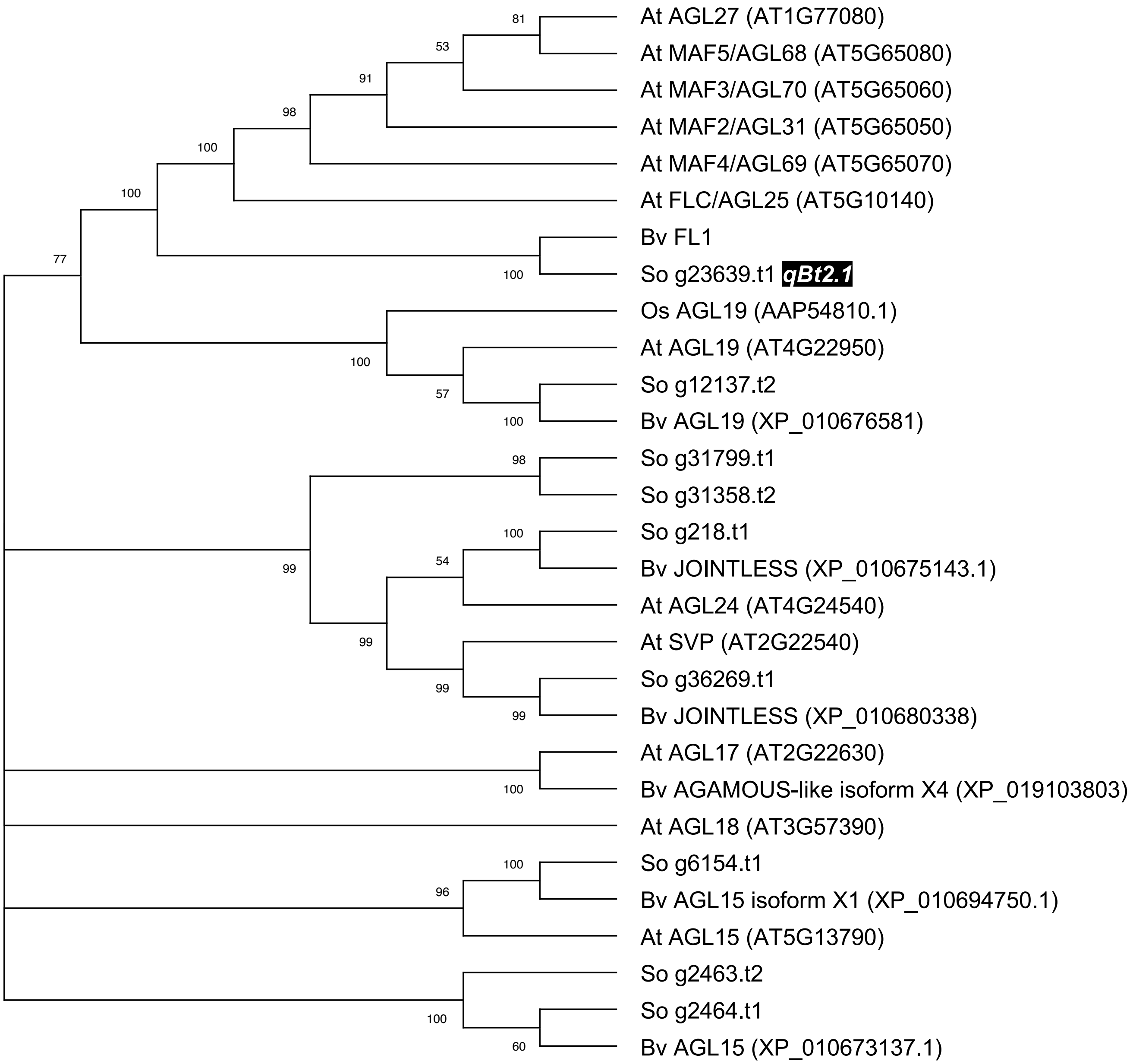

Supplementary Figure S11. Neighbor-joining phylogenetic tree of the amino acid sequences of AGAMOUS-like MADS-box proteins

Plant species are represented by two letters as follows: So, *Spinacia oleracea*; Bv, *Beta vulgaris*; At, *Arabidopsis thaliana*; Os, *Oryza sativa*. Identifiers beginning with “g” indicate the gene IDs (abbreviated forms) of SOL_r1.1a. Gene identifiers in public databases are given in parentheses.

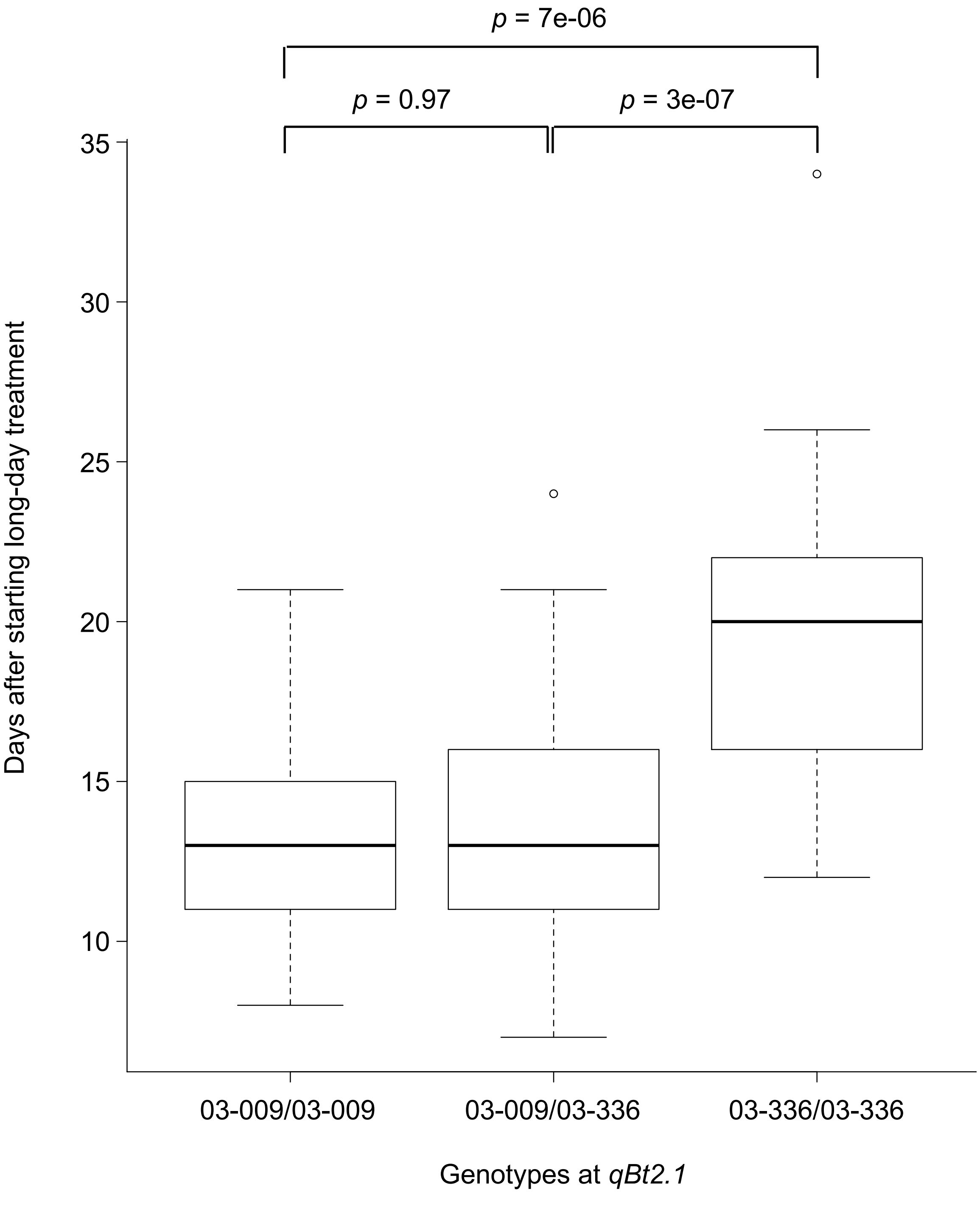

Supplementary Figure S12. Box plot comparing bolting times among genotypes at *qBt2.1* in the F_2 progeny plants from the cross between 03-009 and 03-336

The p-values of the Tukey tests are shown on the plot. Homozygotes for the 03-009 allele (03-009/03-009) and the heterozygous plants (03-009/03-336) bolted significantly earlier than homozygotes for the 03-336 allele (03-336/03-336), whereas there was no significant difference between the 03-009/03-009 and 03-009/03-336 plants. These results suggest that the 03-009 allele may encode a bolting promotor.

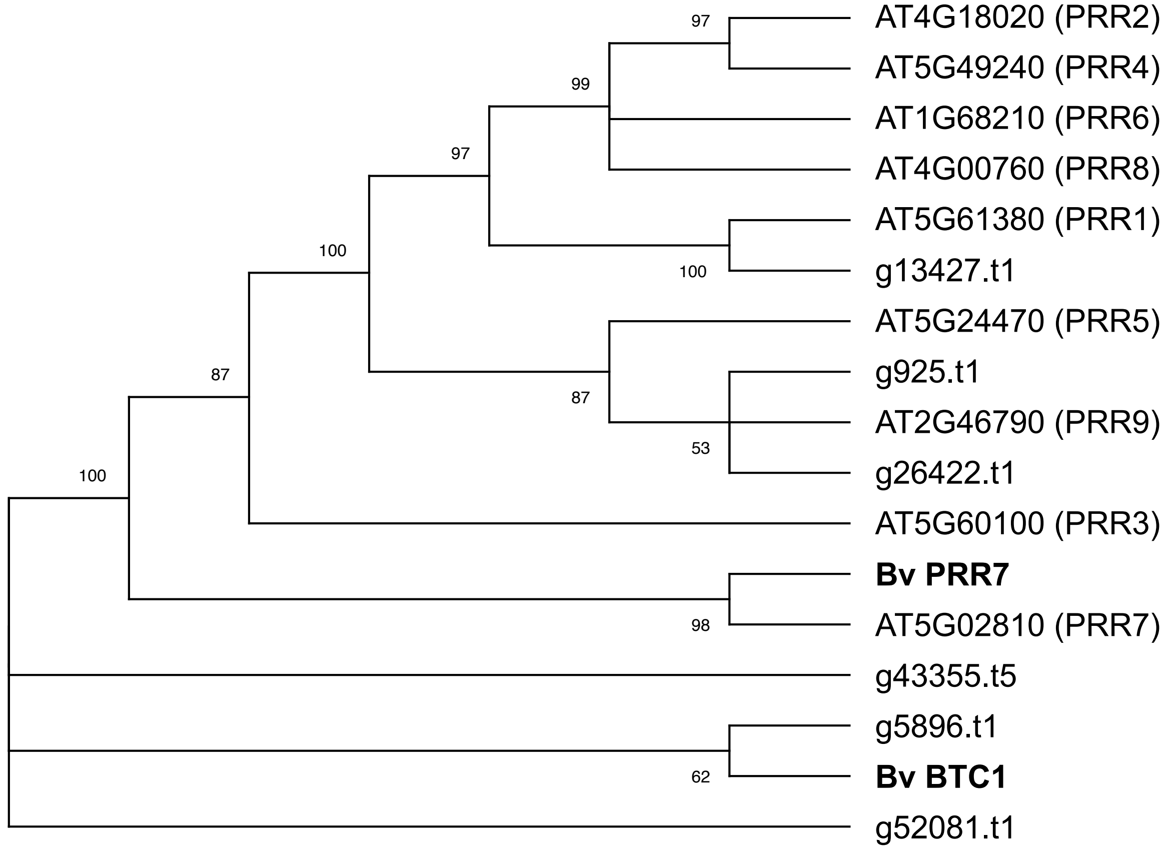

Supplementary Figure S13. Neighbor-joining phylogenetic tree of pseudo-response regulators (PRR) from Arabidopsis, spinach and sugar beet Identifiers beginning with “g” and “AT” indicate the gene IDs of SOL_r1.1 (abbreviated forms) and Arabidopsis locus IDs, respectively. Identifiers shown in bold represent sugar beet (*Beta vulgaris*) PRR proteins.

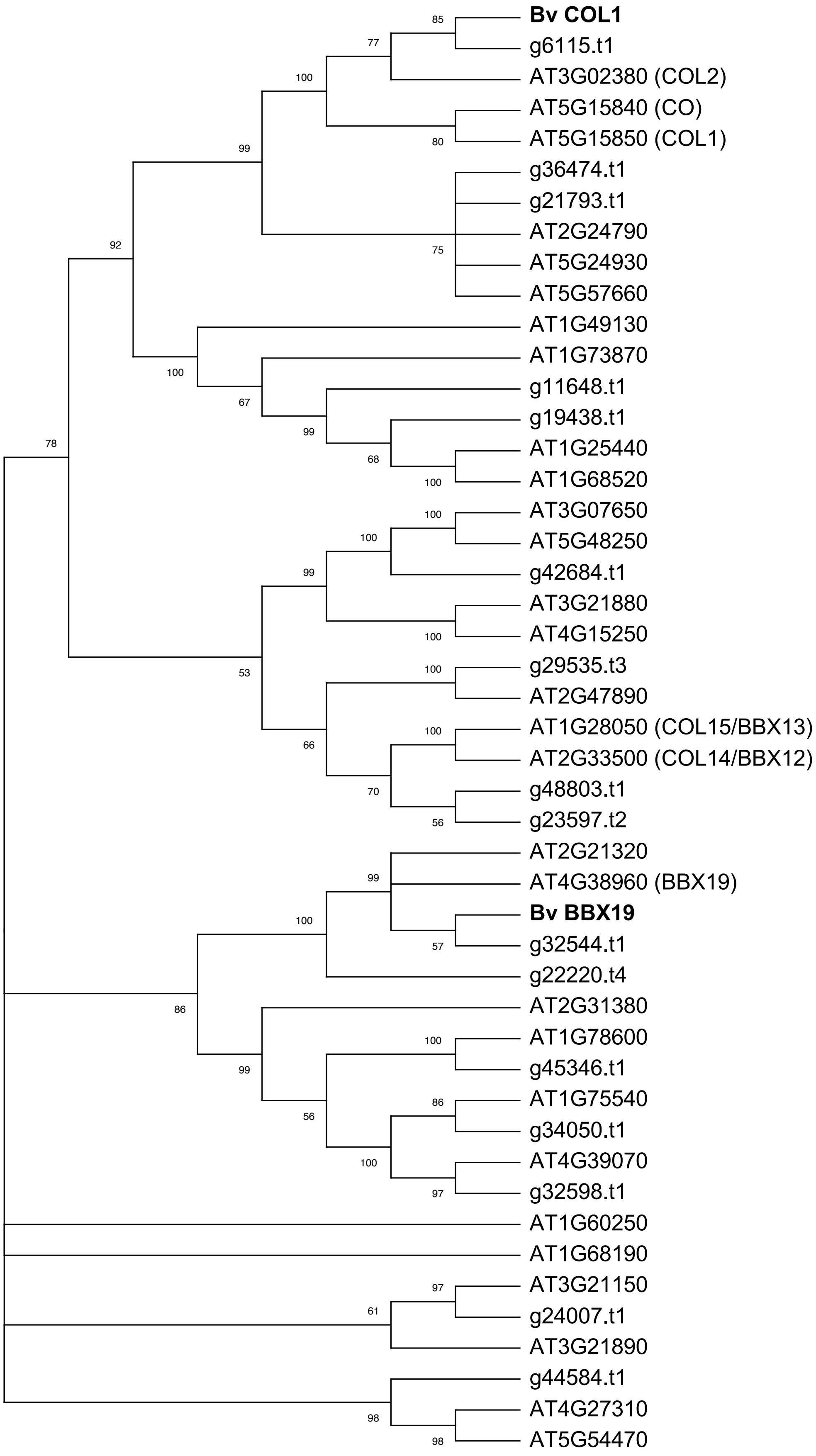

Supplementary Figure S14. Neighbor-joining phylogenetic tree of B-box (BBX) domain proteins from Arabidopsis, spinach and sugar beet Identifiers beginning with “g” and “AT” indicate the gene IDs (abbreviated forms) of SOL_r1.1 and Arabidopsis locus IDs, respectively. Identifiers shown in bold represent flowering time regulators in sugar beet.
